# Compartment‐guided assembly of large‐scale molecular models with *Bentopy*

**DOI:** 10.1002/pro.70480

**Published:** 2026-02-12

**Authors:** M. S. S. Westendorp, J. A. Stevens, C. M. Brown, A. C. Dommer, T. A. Wassenaar, B. M. H. Bruininks, S. J. Marrink

**Affiliations:** ^1^ Groningen Biomolecular Sciences and Biotechnology Institute University of Groningen Groningen The Netherlands; ^2^ Knowledge Center Biobased Economy Hanze University for Applied Sciences Groningen The Netherlands

**Keywords:** all‐atom, coarse‐grained, integrative modeling, Martini, molecular dynamics, model construction, whole‐cell simulations

## Abstract

Molecular dynamics simulations of whole cells and organelles aim to transform cell biology by providing a molecular view into the crowded cellular environment. However, constructing suitable starting configurations remains a major bottleneck due to their size and complexity. We present *bentopy*, a workflow for building cellular‐scale models ready for simulation. *Bentopy* uses a voxel‐based spatial representation to efficiently pack molecules into arbitrarily complex compartments. The modular design facilitates the assembly of molecular models containing millions of molecules in minutes on standard workstations. We demonstrate *bentopy* through three experimentally informed models spanning multiple biological scales. A JCVI‐Syn3A whole‐cell model combines existing membrane and chromosome structures with diverse cytosolic components. A mitochondrion model integrates cryo‐electron tomography maps with spatially resolved proteomics and metabolomics data. An atomistic SARS‐CoV‐2 virion inside a respiratory aerosol demonstrates *bentopy*'s capability to pack at both atomistic and coarse‐grained resolution. Together, these applications establish *bentopy* as a workflow for constructing simulation‐ready models at the cellular scale.

## 1 INTRODUCTION

Recent advances in experimental techniques are revealing cellular structure and organization at unprecedented resolution. Cryo‐electron tomography (cryo‐ET) resolves organelle architectures at nanometer scales (Kühlbrandt, 2022; Maurer et al., 2025; McCafferty et al., 2024; Nogales & Mahamid, 2024), while super‐resolution microscopy and spatial proteomics map molecular distributions within cells (Aslam et al., 2017; Guo et al., 2025; Gwosch et al., 2020). These experimental techniques, enhanced by computational methods for data analysis and molecular modeling (AlQuraishi, 2021; Jumper et al., 2021), provide an increasingly complete picture of cellular organization across scales.

Molecular dynamics (MD) simulations can transform static experimental snapshots into dynamic models. Cellular processes occur in crowded (Alfano et al., 2024; Samuel Russell et al., 2023) and compartmentalized (Bar‐Peled & Kory, 2022; Diekmann & Pereira‐Leal, 2013) environments where spatial organization influences molecular interactions (Guin & Gruebele, 2019). Capturing these contexts requires integrating experimental data from multiple modalities, including structural and compositional data, into a molecular simulation workflow (Brown & Marrink, 2024).

Specialized building tools have facilitated MD simulations of individual biological components such as membranes (Im & Khalid, 2020; Marrink et al., 2019; Trebesch et al., 2025), proteins (Borges‐Araújo et al., 2023; Goossens & De Winter, 2018; Rizzuti, 2022), and nucleic acids (Pérez et al., 2012; Yoo et al., 2020). Studying these components within their native cellular contexts, such as chromosomes within whole cells (Gilbert et al., 2023; Stevens et al., 2023), viruses within aerosol droplets (Dommer et al., 2023), or proteins within the cytosol (Yu et al., 2016), requires assembling multi‐component models at cellular scales. Pioneering work by Goodsell and colleagues has demonstrated the power of cellular‐scale molecular visualization, creating impressive models that highlight the crowded complexity of cellular interiors (Johnson et al., 2015; Maritan et al., 2022). However, MD simulations impose additional requirements on the starting configuration: strictly preventing steric clashes, placing molecules in near‐equilibrium distributions, and integration with existing pipelines.

Several tools address aspects of large‐scale system construction. PACKMOL excels at packing molecules into analytically defined regions (spheres, boxes, cylinders) but lacks support for complex geometries (Martínez et al., 2009; Soñora et al., 2021). Moreover, its packing protocol is optimized for dense systems with a simple spatial distribution, but does not scale efficiently to cellular dimensions. CellPACK generates full‐scale cellular models using computational approaches similar to those we employ here, but models typically require substantial additional processing to prepare them for MD simulations (Goodsell & Autin, 2024; Johnson et al., 2015; Maritan et al., 2022). CHARMM‐GUI provides comprehensive web‐based workflows for constructing protein and polymer systems with simulation inputs, but web‐based operation limits integration into automated modeling pipelines and high‐throughput workflows (Jo et al., 2008; Kern et al., 2024). While these tools address individual requirements for cellular‐scale MD model construction, no single framework integrates spatial complexity, efficient packing, and simulation‐ready output generation.

To resolve these issues, we present *bentopy*, a modular workflow for constructing cellular‐scale MD models. The name draws from the Japanese bento box, reflecting how *bentopy* packs molecular components neatly into compartments within a simulation box. *Bentopy* uses a voxel‐based spatial representation to efficiently pack molecules within complex spatial geometries. The workflow separates spatial definition, molecule placement, and simulation input generation, enabling rapid model iteration as experimental data evolves. *Bentopy* integrates directly with MD workflows, particularly the coarse‐grained Martini ecosystem (Marrink et al., 2023), while also supporting atomistic force fields.

We demonstrate *bentopy* through three experimentally informed models, involving bacterial, organellar, and viral scales: (i) A JCVI‐Syn3A whole‐cell model combining large‐scale membrane and chromosome structures with a diverse cytosolic composition at physiological density, (ii) A mitochondrion model integrating a cryo‐ET map with spatially resolved proteomics and metabolomics data, and (iii) An atomistic SARS‐CoV‐2 virion inside a respiratory aerosol containing more than a billion atoms, demonstrating the force field–agnostic workflow. These applications establish *bentopy* as a workflow for creating digital twins, bridging the gap between experimental data and computational models.

## 2 DESIGN AND IMPLEMENTATION

**FIGURE 1:**
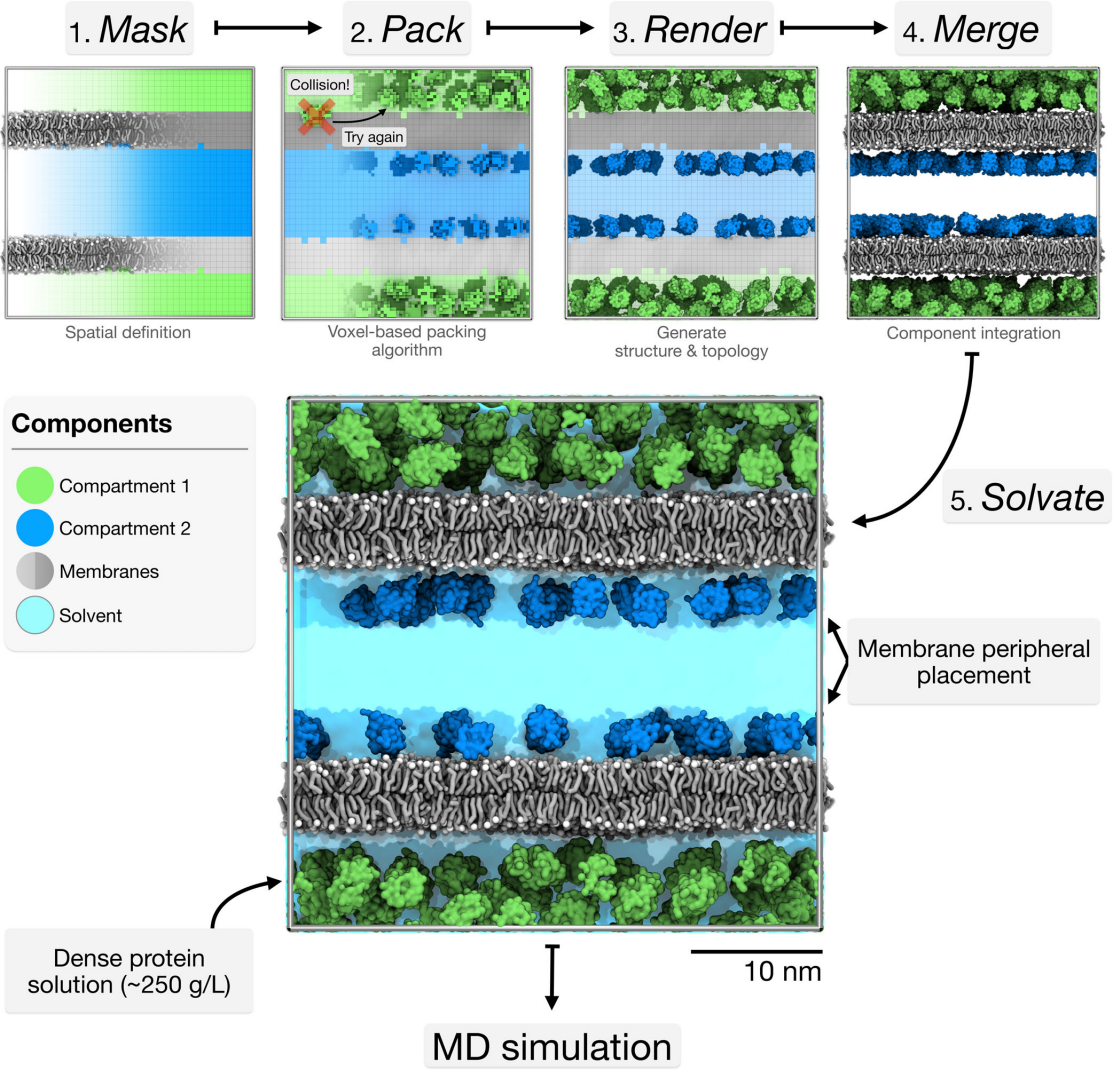
*Bentopy* workflow demonstrated with a double‐bilayer model. Overview of the *bentopy* workflow steps: 1. spatial masking defines the compartments, 2. voxel‐based packing places the proteins, 3. rendering generates simulation input files (coordinates and topology), 4. merging integrates components, and 5. solvation finalizes the simulation‐ready model. We demonstrate compartment‐specific packing via a dense protein solution (~250 g/L, green compartment) and membrane‐peripheral proteins (blue compartment). See the *bentopy* tutorial for a step‐by‐step construction guide (Martini Force Field Initiative, 2026).

The *bentopy* workflow separates model construction into independent operations that users combine based on model complexity. Three core operations handle molecule placement: spatial definition (*mask*), constraint‐aware packing (*pack*), and coordinate generation (*render*). Model assembly (*merge*) and solvation (*solvate*) complement the workflow for cellular systems. This modular design enables iterative model refinement, allowing users to update molecule concentrations, exchange protein structures, or incorporate new experimental data by rerunning only the affected steps rather than reconstructing the entire model. Such incremental modification is valuable for multi‐component systems and becomes essential for large‐scale cellular models where construction difficulty scales with system complexity. The modular design also provides a foundation for extending the workflow as new challenges emerge in cellular‐scale modeling and as the ecosystem of MD simulation tools evolves. We aim to conduct the development of the *bentopy* project according to modern software development recommendations (Amaro et al., 2025). Below, we describe the algorithmic approaches underlying each operation, emphasizing the capabilities they enable for cellular‐scale model construction (Figure 1).

### 2.1 *Mask*: Voxel‐based spatial masking

At the core of *bentopy*'s capabilities is the notion of volumetric masks. These masks represent three‐dimensional space as a regular voxel grid, where voxels encode spatial information for guiding molecule placement. The voxelization transforms complex geometries into computationally efficient spatial representations that enable the efficient packing algorithm. The *mask* operation generates these spatial definitions from existing molecular structures using MDVContainment (Bruininks & Vattulainen, 2025), a compartment detection algorithm that identifies and labels compartments within complex geometries under periodic boundary conditions. Compartment detection is essential for the packing workflow, as different regions require different molecular compositions. Cytoplasmic proteins, for instance, must be confined within the enveloping cell membrane and excluded from the extracellular space.

Masks are stored as compressed arrays, enabling efficient storage and access between operations. The modular workflow design also allows users to provide externally generated masks directly, complementing or replacing the automated compartment detection. This flexibility allows integration of spatial information from experimental sources such as cryo‐ET.

### 2.2 *Pack*: Constraint‐aware molecule placement

Building cellular environments requires satisfying multiple competing constraints: physiological concentrations, compartment boundaries, preventing molecular overlaps, and capturing experimentally informed spatial distributions. The *pack* operation addresses this through a constraint‐aware algorithm configured by an input file specifying molecular structures, target concentrations or copy numbers, and placement rules.

The algorithm prioritizes efficient packing at large scales through voxel‐based collision detection. Molecules are ranked by a packing difficulty heuristic based on geometric moment of inertia, prioritizing difficult‐to‐pack structures first. For each placement attempt, the algorithm samples a random position and rotation, then voxelizes the molecule. Placements are accepted if they satisfy the specified spatial constraints and do not overlap with previously placed molecules. If a placement fails, the algorithm retries a configurable number of times with different positions and orientations before proceeding to the next molecule. The packing procedure is fully reproducible when a random seed is specified. Placement rules provide fine‐grained spatial control over molecule placement. Rules restrict molecules to specific compartments, enforce distance requirements to reference structures, or limit placement to analytically defined spatial regions (e.g., spheres or cuboids). Each rule translates to a voxel mask defining valid placement locations. Multiple rules combine through Boolean operations to capture complex spatial distributions, for example, packing membrane‐peripheral proteins throughout the cytosol while biasing them toward the membrane.

The packing operation outputs a placement list storing molecular identities, positions, and rotations in a compact instanced file format. This lightweight representation decouples the computationally expensive packing step from the coordinate file generation, enabling efficient storage and flexible post‐processing.

### 2.3 *Render*: Coordinate and topology generation

The *render* operation converts placement lists into coordinate and topology files compatible with MD simulation engines. For each entry in the placement list, the molecular coordinates are transformed according to the recorded positions and rotations and then written to the output structure file. The topology file is automatically written during rendering by logging the molecule types and their counts. This approach provides flexibility for model inspection and debugging.

The *render* operation supports multiple output resolutions, from the full detail needed for simulation to simplified representations for visualization. Users can generate spatially restricted outputs by rendering only structures within specified coordinate ranges. This granular control over the output structure file is particularly valuable when working with billion‐particle models where full visualization may be computationally prohibitive for quick validation and iteration. Currently, *bentopy* by default generates GROMACS input files, though extension to support other MD software is planned for future development (Abraham et al., 2015). Users can already connect *bentopy* to other MD engines through existing file conversion tools (MacCallum et al., 2023; Shirts et al., 2017).

The three core operations above handle molecule placement within defined spatial constraints. Constructing complete simulation‐ready cellular models requires two additional steps: integrating packed molecules with template structures, and adding solvent and ions. *Bentopy* also provides specialized tools optimized for these large‐scale operations.

### 2.4 *Merge*: Model assembly

The *merge* operation integrates packed molecules with reference structures that established the initial packing constraints, concatenating coordinate files into unified models. This step is essential when packing occurs within pre‐existing geometries, for example, packing proteins inside a lipid vesicle that was separately constructed. The operation implements optional residue relabeling, assigning residue names by functional context rather than chemical identity. At cellular scales, organization by compartment (membrane, cytoplasm, nucleus) often provides more meaningful visualization and analysis than tracking individual molecular species. This functional grouping simplifies working with models where the same molecule appears in different cellular contexts with distinct biological roles.

### 2.5 *Solvate*: Large‐scale solvation

Solvation represents a computational bottleneck for building cellular‐scale models, as typically 60%–70% of the final model consists of water molecules. While biomolecules represent the functionally interesting components, the vast majority of particles are added during solvation, creating memory overhead and I/O bottlenecks that conventional tools struggle to handle efficiently.

The *solvate* operation follows the canonical approach of tiling an equilibrated solvent box template across the solute structure, but addresses scalability challenges through a bitmap strategy. Rather than storing all solvent molecules in memory during solvation, the algorithm spatially partitions the structure into grid regions and uses a placement map that tracks only the occupied/available states of each potential solvent position. This reduces memory requirements while enabling efficient collision detection through spatial mapping rather than brute‐force distance calculations. The tool provides control over solvent–solute cutoff distances, enabling users to optimize solvation quality by preventing both vacuum bubble artifacts and solvent–solute overlap errors.

Ion placement and molecular substitutions (such as antifreeze particles for the Martini 2 force field) integrate into the solvation process rather than requiring separate post‐processing. Users can specify substitution quantities through molar concentrations for physiological conditions, percentage compositions for specific ratios, or absolute particle numbers, providing flexibility across different use cases. This integrated approach eliminates computational bottlenecks while ensuring proper charge neutralization and physiological ionic strength in the final solvated model.

In the following sections we will present the capabilities of the *bentopy* workflow by showcasing a set of new complex molecular models. These models span from cell to cell organelles to aerosol particles. All models were constructed on standard workstation hardware, demonstrating the accessibility of the workflow. The validity of these models has been verified with a short MD simulation.

## 3 RESULTS

### 3.1 JCVI‐Syn3A minimal cell

The JCVI‐Syn3A minimal cell represents the simplest known living bacterium with only 452 protein‐coding genes (Breuer et al., 2019; Pelletier et al., 2021). This makes it an ideal test case for demonstrating whole‐cell modeling capabilities. Building on previous work by Stevens et al. (2023), we constructed a 200 nm diameter coarse‐grained model using the Martini 2 force field. This 200 nm diameter represents approximately half the size of a typical Syn3A cell, maintaining the compositional complexity while improving computational tractability. The model integrates the placement of a large and diverse set of biomolecules with pre‐built chromosome and envelope structures, validated in previous work (Gilbert et al., 2023; Stevens et al., 2023). The final model includes 7193 proteins, 554 RNA structures, and 34,693 metabolites restricted to species with available Martini 2 parameterizations. The molecular composition and concentration are based on experimental measurements including proteomics and metabolomics (Breuer et al., 2019; Thornburg et al., 2022).

*Bentopy* enables seamless integration with pre‐built molecular models. We used the *merge* operation to combine the chromosome and cell envelope structures into a single model template. From this, we generated a packing mask using *bentopy*'s automated compartment detection, which identified the intracellular space within this model template. We used a 0.5 nm resolution voxel mask representing 10,000,000 nm^3^ of accessible cytoplasmic volume. We generated a second mask representing the cell envelope for biasing molecular placement with respect to the membrane.

Proteomics annotations distinguish soluble proteins from membrane‐peripheral proteins. Rather than distributing all proteins uniformly, we implemented spatial rules based on membrane proximity. Membrane‐peripheral proteins were constrained to within 15 nm of the envelope and cytosolic proteins were allowed to occupy the full intracellular volume. Incorporating the experimental data ensured the model captures the realistic spatial organization of the cell as closely as possible.

**FIGURE 2:**
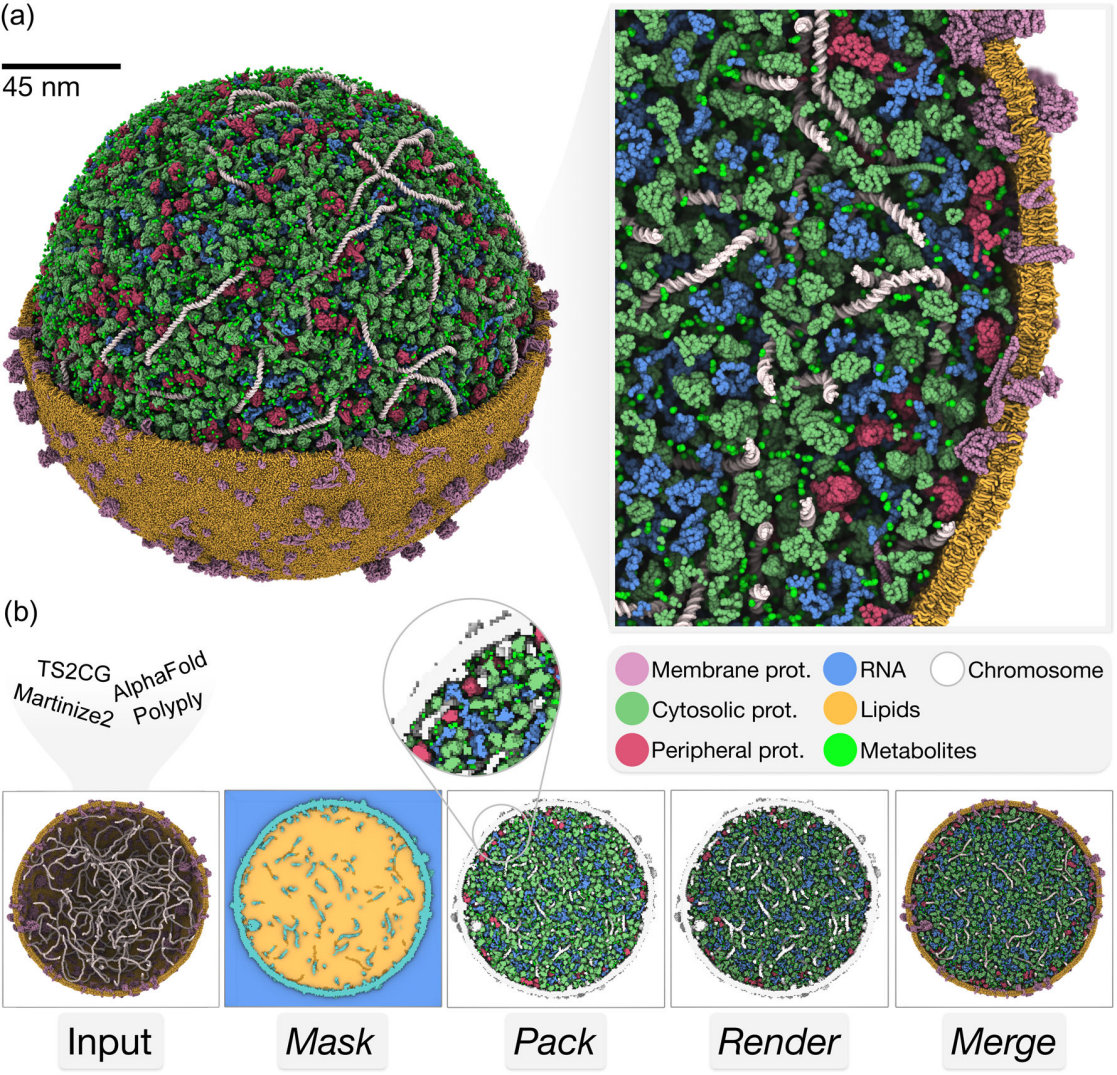
JCVI‐Syn3A minimal cell. (a) JCVI‐Syn3A minimal cell model with an inset highlighting the compositional complexity and density of the built cytosol model. The model integrates seven molecular components: Chromosome (white), lipids (orange), membrane proteins (pink), cytosolic proteins (green), peripheral membrane proteins (red), metabolites (lime), and RNA (blue). (b) *Bentopy* workflow steps: Starting from pre‐built JCVI‐Syn3A chromosome and membrane models, followed by spatial masking, molecular packing, and rendering. The final panel shows the complete model integrating cytosolic components with pre‐built membrane and chromosome models.

Packing completed in 4 min, successfully placing 99.7% of requested molecules. Only the largest RNA polymers remained unpacked due to their elongated geometry and would need to be included from the beginning. The unsolvated system comprises 16 million coarse‐grained beads across more than a thousand distinct molecular species (Figure 2). A detailed model composition is provided in Table S1. After solvation with Martini water and 150 mM NaCl, it reaches 87 million beads, equivalent to around 1 billion atoms. The model achieves a cytosolic density of 520 g/L, calculated from the total dry mass of cytosolic components divided by the accessible volume. This value is close to the experimental range for bacterial cytoplasm, validating the physiological realism of the model composition (Ellis & Minton, 2003).

To validate the model, we performed an MD simulation using the standard Martini 2 protocol as explained in the methods section. The solvated model required regular energy minimization and brief equilibration before we could perform a stable 1 ns production simulation.

### 3.2 Mitochondrial compartments

To demonstrate *bentopy*'s compartment‐aware packing, we constructed a coarse‐grained model of the compartments of a mitochondrion based on an experimentally resolved cryo‐ET structure. Mitochondria exemplify intracellular compartmentalization, with dramatically different molecular compositions. The compartments are formed by two non‐intersecting membranes separating the inner mitochondrial matrix and the intermembrane space (IMS). The latter we here take to include the cristae volume (lumen). The mitochondrial membrane architecture is essential for organelle function, with proteins such as ATP synthase dimers organizing highly curved cristae structures (Daumke & van der Laan, 2025; Kühlbrandt et al., 2025). Building molecular models directly from experimental structural data represents a key direction in integrative modeling. The combination of compartment‐specific compositions with complex experimental geometry makes mitochondria an ideal demonstration of the *bentopy* workflow.

**FIGURE 3:**
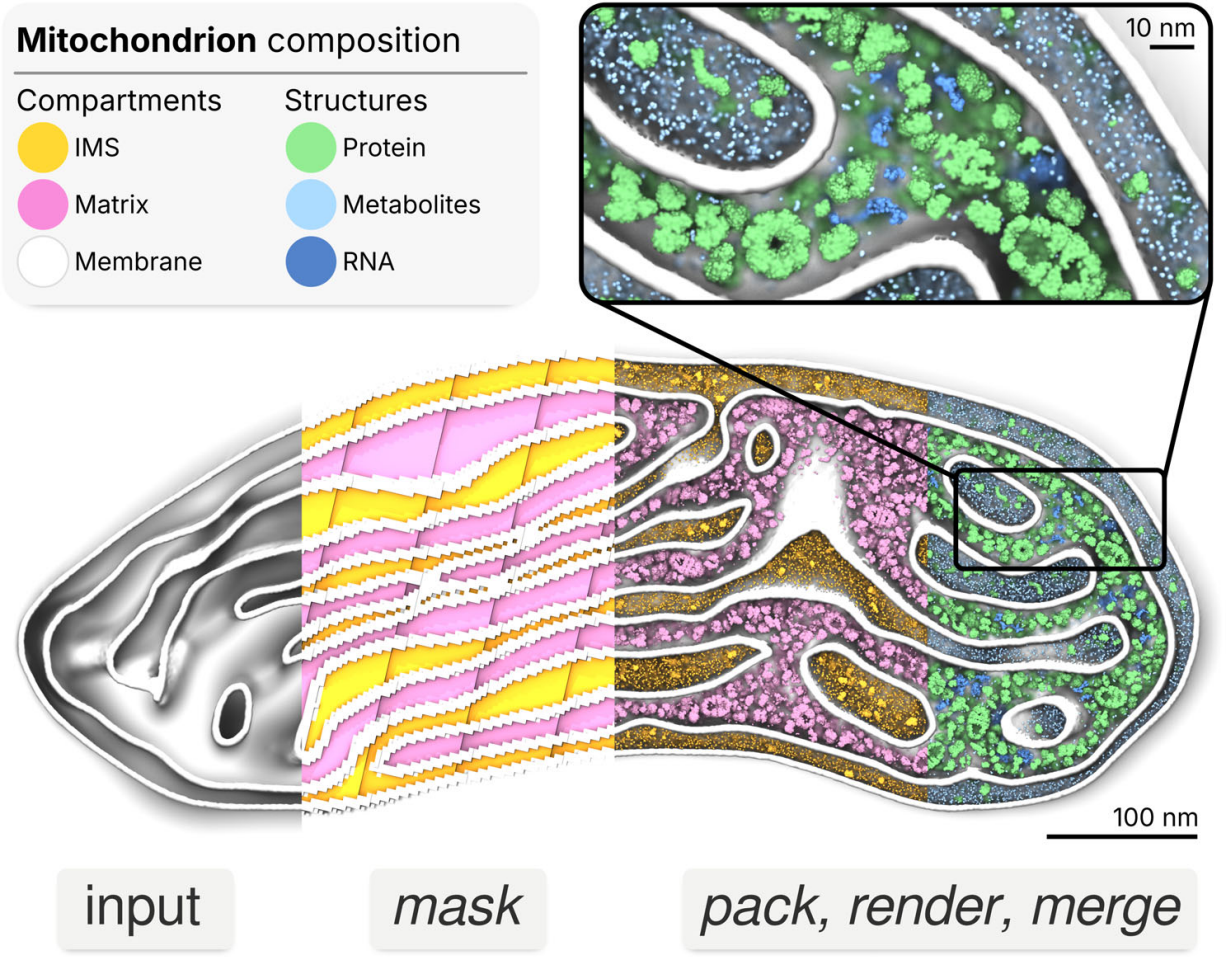
Model of mitochondrial compartments. The assembly of the mitochondrial model based on an experimentally informed membrane structure (white), from an empty structure (left) into a mask representation of the IMS (yellow) and matrix (pink) compartments represented by 3 nm voxels. Structures are packed into their assigned compartments based on the mask. In the last section and the magnified inset, structures are colored by kind: Proteins (green), RNA (dark blue), metabolites (pale blue).

A cryo‐ET map of a mouse neuron mitochondrion (Garcia et al., 2019) yielded the membrane geometry as a triangulated surface mesh. Using TS2CG (Pezeshkian et al., 2020; Schuhmann et al., 2025), we converted this mesh into a lipid bilayer spanning 344 × 796 × 416 nm^3^. This membrane was solely used to define the mitochondrial compartments for cytosolic packing, that is, we did not attempt to model the full complexity of the mitochondrial membranes. Both the IMS and matrix compositions were determined from human mitochondrial proteomics and metabolomics data (Chen et al., 2016; Morgenstern et al., 2021). We selected the 10 most abundant soluble proteins from the MitoCoP dataset (Morgenstern et al., 2021) for each compartment, obtaining structures from the PDB (Berman et al., 2000) or AlphaFold3 (Abramson et al., 2024). Protein concentrations were scaled to rough estimates of the total protein density in both the IMS and matrix (Chen et al., 2016; Morgenstern et al., 2021). In the final model, the protein densities are 26 g/L in the IMS and 239 g/L in the matrix. The resulting 9‐fold density contrast is immediately apparent visually (Figure 3), distinguishing the protein‐dense metabolic matrix and the less‐crowded IMS primarily involved in transport. The metabolome was restricted to species with available Martini 2 parameterizations (Monticelli et al., 2008; Sousa et al., 2021), yielding total concentrations of 114 mM in the IMS and 7 mM in the matrix, a 16‐fold difference, reflecting their distinct metabolic environments.

We generated 0.5 nm resolution voxel masks using the compartment detection capabilities, distinguishing the IMS (12 × 10^6^ nm^3^) and matrix (16 × 10^6^ nm^3^) volumes. Each compartment was packed with its experimentally determined composition. The packing completed in under 4 min, placing approximately 17 thousand proteins and 900 thousand metabolites across both compartments.

The unsolvated model comprises 122 million Martini 2 CG beads across 56 distinct molecular species. A detailed model composition is provided in Table S2. Solvation with Martini water, 150 mM NaCl, and charge‐neutralizing ions increased the system to over 985 million beads, representing more than 10 billion atoms at atomistic resolution. To validate the model, we performed an MD simulation using the standard Martini 2 protocol as explained in the methods section. The solvated model proved stable during a 1 ns run, after energy minimization and brief equilibration with a reduced timestep.

### 3.3 SARS‐CoV‐2 aerosol

To demonstrate the force field and resolution‐agnostic nature of the *bentopy* workflow, we constructed an all‐atom model of a SARS‐CoV‐2 virion embedded in a respiratory aerosol based on earlier work by Dommer et al. (2023). This billion‐atom work represents one of the largest all‐atom MD models to date, making it an ideal benchmark for evaluating *bentopy*'s capacity to build large multi‐component models at atomistic resolution.

The model consists of a complete SARS‐CoV‐2 virion envelope surrounded by deep lung fluid, forming an aerosol particle placed in a 300 × 300 × 300 nm^3^ periodic box. The lung fluid composition is based on artificial saliva and surrogate deep lung fluid recipes (Vejerano & Marr, 2018; Walker et al., 2021) and includes phospholipids (DPPC, DPPG), cholesterol, ions (Ca^2+^, Mg^2+^, K^+^, Na^+^, Cl^−^), human serum albumin, and mucin glycoproteins. The mucin components consist of three long glycopolymers, each ∼55 nm in length, with full O‐glycosylation patterns based on experimentally determined protein and glycosylation sequences (Kearns et al., 2024).

The *bentopy* workflow used the SARS‐CoV‐2 virion structure as the model template (Dommer et al., 2023). We generated a 0.5 nm resolution voxel mask from this structure to define the excluded volume for the packing procedure. To reproduce experimentally observed aerosol sizes (Ke et al., 2020), we constrained packing to a 270 nm diameter sphere encompassing the virion, using *bentopy*'s packing rules. *Bentopy* successfully packed all components collision‐free, completing the packing in 9 seconds. The model contains 23,566 molecules totaling 67 million atoms before solvation. A detailed model composition is provided in Table S3. The solvation with TIP3P water was restricted to the spherical aerosol region, leaving vacuum outside the aerosol. After neutralization with Na^+^ ions, the total atom count reached approximately 1 billion atoms.

**FIGURE 4:**
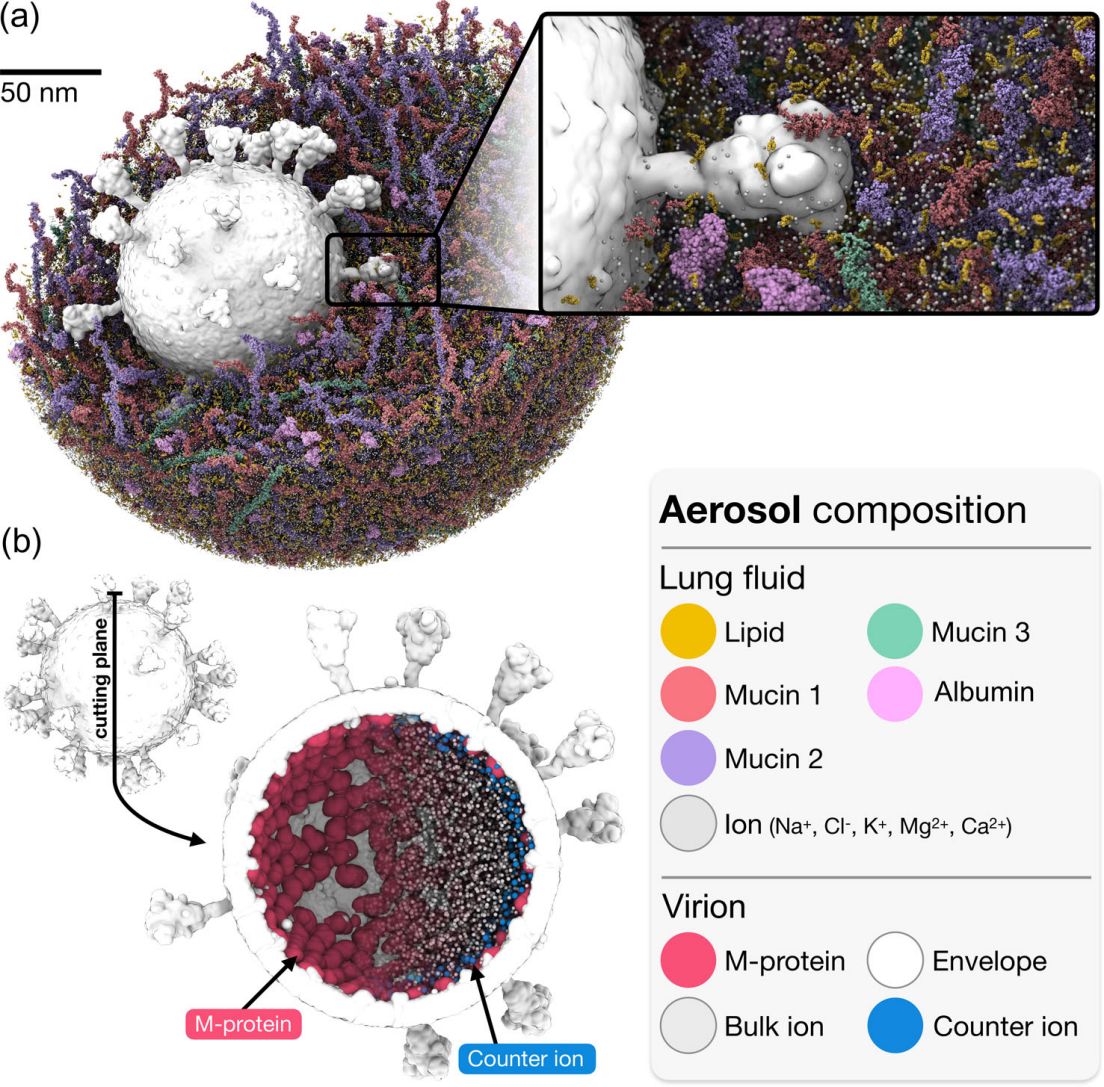
SARS‐CoV‐2 virion inside a respiratory aerosol. (a) All‐atom model of the SARS‐CoV‐2 virion embedded in a 270 nm diameter spherical aerosol of deep lung fluid. The inset shows the virion‐aerosol interface in detail. (b) Cross‐section of the virion highlighting spatially informed placement of Na^+^ counter‐ions (purple spheres) within 5 nm of the negatively charged M‐protein dimers (red) in the viral membrane (white).

*Bentopy* enabled several key improvements to the original aerosol model (Dommer et al., 2023). Most notably, the full‐length mucin polymers (up to 55 nm in length) were packed within the aerosol volume to 100% of the targeted mucin concentration. This contrasts with the original workflow which packed mucins in smaller boxes, followed later by assembling them into the larger model, resulting in the final structure containing fewer mucins than intended (Dommer et al., 2023). *Bentopy* significantly simplifies the building workflow and reduces the risk of packing artifacts. Additionally, we used *bentopy*'s placement rules to position 5695 Na^+^ counter‐ions within 5 nm of the M‐protein dimers inside the virion, compensating for their negative charge. This targeted neutralization results in an initial configuration closer to equilibrium, improving the model stability and reducing the required equilibration time (Figure 4).

To validate the model, we performed an energy minimization using CHARMM36m (Huang et al., 2017). However, computing long‐range electrostatic interactions using Particle Mesh Ewald (PME) in GROMACS for a system this size proved prohibitively memory‐intensive, rendering subsequent equilibration and production runs infeasible. The successful minimization demonstrates the clash‐free structure of the built model, with the simulation limitation arising solely from technical constraints of the hardware and software.

## 4 DISCUSSION

Constructing MD models at cellular scales requires integrating diverse experimental data into simulation‐ready models containing millions of molecules. Existing tools handle detailed molecular assemblies at limited scales, construct only specific structural elements, or generate representative models that are not guaranteed simulation‐ready. *Bentopy* addresses this gap through a workflow optimized for cellular‐scale multi‐component models, producing MD‐compatible outputs that integrate directly with existing simulation ecosystems. The design prioritizes both computational efficiency and rapid iteration, enabling the compositional refinement essential to integrative modeling. *Bentopy* transforms cellular model construction into a reproducible, data‐driven workflow. Researchers can modify concentrations, update molecular structures, or adjust placement rules and regenerate the complete model in minutes rather than days.

The three showcase models demonstrate *bentopy*'s versatility across biological scales and force fields. The minimal cell captures compositional complexity with over 1000 unique molecular species, the mitochondrion demonstrates compartment‐guided packing from spatially resolved experimental data, and the aerosol establishes atomistic model construction at large scales. These applications span system sizes from millions to billions of particles with construction times of seconds to minutes.

Iterative refinement is fundamental to integrative modeling; as new experimental data become available, models must evolve alongside these measurements. While manageable for small systems, this can become prohibitively slow at cellular scales, making systematic exploration of compositional space impractical. Furthermore, having a condensed description of the model composition that can be used to quickly and reproducibly rebuild the model is crucial. *Bentopy* enables the construction of these “living models” that continuously incorporate new findings rather than remaining static snapshots, making iterative integration practical even for billion‐particle systems.

*Bentopy* constructs cellular‐scale models on standard workstations without GPU acceleration. The voxel‐based collision detection and bitmap solvation algorithm achieve computational efficiency through spatial data structures that speed up costly distance calculations. Our mitochondrion model (containing ~1 billion particles) was built in under 4 min on a workstation accessible for most research groups. Reproducibility is ensured through strict versioning, standardized file formats and controllable random seeds. The input files provide a complete model description, enabling exact reproduction. Users can share lightweight workflow descriptions rather than multi‐gigabyte coordinate files, with version control tracking model evolution over time. This contrasts with manual construction through ad hoc scripts or web‐based tools where reproducing exact configurations proves challenging.

*Bentopy* generates simulation‐ready outputs that integrate directly with existing MD workflows, currently supporting GROMACS output files. The workflow integrates particularly well with the Martini ecosystem, which enables cellular‐scale simulations through a 4‐to‐1 atom mapping. Our showcase systems leverage this ecosystem integration by using protein models built with Martinize2 (Kroon et al., 2025), membranes from TS2CG (Schuhmann et al., 2025), and polymers from Polyply (Grünewald et al., 2022) which are assembled into complete cellular models with *bentopy*. This means users employ specialized tools for individual components while *bentopy* handles the multi‐component assembly challenge. The modular architecture also supports atomistic simulations, as our SARS‐CoV‐2 aerosol model demonstrates. *Bentopy* is distributed via standard Python package managers (pip, conda) and developed following modern software practices (Amaro et al., 2025).

*Bentopy*'s rigid‐body packing approach suits most cellular components but struggles with extended polymers. For polymer systems, a random walk protocol is better suited to create high‐density polymer simulations. Interfacing the *bentopy* workflow with sophisticated polymer building tools, like Polyply, opens up a promising avenue for facilitating both the efficient packing of multi‐component models for rigid‐globular molecules, while the polymers can be handled with an efficient random walk approach. This leverages the strengths of both methods for constructing multi‐component cellular models. Furthermore, the greedy packing algorithm does not guarantee complete packing to target densities. While backtracking could improve packing completeness, we found it unnecessary for our showcase systems at physiological densities (~300 mg/mL cytoplasm). The stochastic placement typically achieves 95%–100% of target concentrations.

Several extensions would expand *bentopy*'s capabilities and integrate it more deeply with experimental and simulation workflows. Generalizing mask generation to accept experimental data directly, such as cryo‐ET density maps or fluorescence microscopy intensity distributions, would enable data‐driven spatial constraints without intermediate processing. Similarly, supporting mesh‐based geometries for membrane surfaces would facilitate integration with tools like TS2CG for better handling membrane topologies. Connecting *bentopy* to other simulation methods could enable multi‐scale modeling of cellular processes. Kinetic models of metabolism or signaling provide concentration information but lack spatial detail (Macklin et al., 2020; Thornburg et al., 2022). *Bentopy* could generate spatially resolved initial conditions for particle‐based simulations while importing concentration distributions from kinetic models. This integration would bridge cellular systems biology and molecular simulation.

*Bentopy* facilitates integrative modeling where computational models and experimental data develop in parallel. As experimental techniques generate increasingly detailed cellular data, corresponding computational tools must handle this complexity while remaining accessible and reproducible. Our modular, extensible design allows integration of new experimental data types as mask sources and spatial distributions as placement rules. This positions *bentopy* as infrastructure for the emerging field of cellular‐scale molecular modeling.

## AUTHOR CONTRIBUTIONS

**M. S. S. Westendorp:** Software; visualization; writing – original draft; methodology; validation. **J. A. Stevens:** Conceptualization; software; visualization; writing – original draft; methodology; validation. **C. M. Brown:** Writing – review and editing; validation. **A. C. Dommer:** Writing – review and editing; validation. **T. A. Wassenaar:** Writing – review and editing. **B. M. H. Bruininks:** Writing – review and editing; methodology. **S. J. Marrink:** Funding acquisition; supervision; resources; methodology; writing – original draft.

## CONFLICT OF INTEREST STATEMENT

The authors declare no conflicts of interest.

## Supporting information


**Data S1.** Supporting information.

## Data Availability

*Bentopy* is open‐source software distributed under the Apache 2.0 license. The source code, documentation, and installation instructions are available on GitHub (https://github.com/marrink-lab/bentopy). Users can report issues, request features, and contribute to development through the GitHub repository. A comprehensive tutorial demonstrating the bentopy workflow is available on the CGMartini website (https://cgmartini.nl/docs/tutorials/Martini3/Bentopy/). All models presented in this work are available on Zenodo (https://doi.org/10.5281/zenodo.17524049), including input files, placement lists, and final coordinate and topology files for MD simulations.
