# Supplementary material for: Compartment‐guided assembly of large‐scale molecular models with Bentopy
Source: Protein Sci. 2026 Feb 12;35(3):e70480. doi: 10.1002/pro.70480 (PMC12895369; doi:10.1002/pro.70480)
Supplement: Supplementary file 1 — Data S1. Supporting information. [file PRO-35-e70480-s001.pdf]

Supplementary Material

Compartment-Guided Assembly of Large-Scale Molecular Models with *Bentopy*

M.S.S. Westendorp[1#], J.A. Stevens[1#], C.M. Brown[1], A.C. Dommer[1], T.A. Wassenaar[1,2], B.M.H. Bruininks[1], S.J. Marrink[1*]

[1] Groningen Biomolecular Sciences and Biotechnology Institute, University of Groningen, Groningen, The Netherlands
[2] Knowledge Center Biobased Economy, Hanze University for Applied Sciences, Groningen, The Netherlands

# Both authors share equal contributions to this work.
* **Corresponding author:** s.j.marrink@rug.nl

Simulation protocols

Coarse-grained simulations

Coarse-grained MD simulations employed the Martini 2 force field using Gromacs 2024.3 (Abraham et al., 2015; De Jong et al., 2016). Models were first energy-minimized using the steepest descent algorithm, followed by equilibration and production simulations (NPT). The simulations employed reaction-field electrostatics and potential-shift-verlet van der Waals treatment, both with 1.1 nm cutoffs (De Jong et al., 2016). To address potential neighbor list artifacts, recently recommended settings were applied (verlet-buffer-tolerance = -1, rlist = 1.35 nm) (Kim et al., 2023). Temperature was controlled at 300 K using the V-rescale thermostat (Bussi et al., 2007). Isotropic pressure coupling at 1 bar utilized the c-rescale barostat during both equilibration and production simulations, with the compressibility set to 3.0×10^{-4} bar^{-1} (Bernetti & Bussi, 2020). For standard simulations an integration timesteps of 10 fs were used for equilibration, and were increased to 20 fs during production simulations. For the large-scale models a reduced timestep of 10 fs was used during the production simulations to ensure numerical stability.

Atomistic simulations

All-atom MD simulations employed the CHARMM36m force field using Gromacs 2024.3 (Abraham et al., 2015; Huang et al., 2017). Models were first energy-minimized using the steepest descent algorithm, followed by equilibration and production simulations. Electrostatic interactions were treated with particle mesh Ewald (PME) with a 1.2 nm cutoff (Essmann et al., 1995). Temperature was controlled at 300 K using the V-rescale thermostat (Bussi et al., 2007) . No pressure coupling was applied, as the aerosol model represents a droplet in vacuum (NVT). Equilibration simulations also used NVT conditions with position restraints on non-water components to allow solvent to relax around the model. Production simulations proceeded without restraints. Integration timesteps were 1 fs for equilibration and 2 fs for production.

Bentopy workflow

Input file structure

Constructing complex MD models requires specifying molecular compositions, spatial constraints, and placement rules in a reproducible format. Bentopy uses a specialized configuration file (`.bent`) to define all parameters for the packing algorithm. The input file comprises five sections (Figure S1). The *General* section sets global parameters, including the random seed for reproducible placement. The *Space* section defines the simulation box dimensions, voxel resolution, and spatial compartments, which can be specified using voxel masks or analytical definitions. The *Includes* section the structure topology files to include in the system topology created by *render*. In the *Compartments* section, compartments are defined by importing them from space mask files, by using analytical shapes, or through combining different compartments. The *Segments* section defines individual molecular species for packing.

Each segment specifies the molecular structure file, target copy number or concentration, and the compartments in which it may be placed. Segments can be assigned to single or multiple compartments, enabling both compartment-specific distributions and combined placement across regions.

The input file structure separates spatial definition from molecular composition, enabling easy iteration on model compositions without regenerating spatial masks. Complete input files for all models presented in this work are available in the supplementary data repository. Detailed documentation on input file structure and all available parameters is provided in the GitHub wiki (*Marrink-Lab/bentopy*, 2026).

```
[ general ]
title "Cell"
seed 0
max-tries-mult 2000

[ space ]
# All sizes and distances are given as nanometers.
dimensions 214, 214, 214
resolution 0.5

[ includes ]
"martini_v2.1_dna.itp"
"martini_v2.0_ions.itp"
"chromosome.itp"
"cytosolic_proteins.itp"
"rna.itp"
"metabolites.itp"

[ compartments ]
cytosol from "cytosolic_space.npz"
membrane from "membrane.npz"
close-to-membrane within 20 of membrane
cytosol-close-to-membrane combines
    cytosol and close-to-membrane

[ segments ]
002_monomer:cyt 19
    from "structures/proteins/002_monomer.gro"
    in cytosol
~~~~~~~~
146_monomer:mp   2
    from "structures/proteins/146_monomer.gro"
    in cytosol-close-to-membrane
~~~~~~~~
ATP           6711
    from "structures/metabolites/ATP.gro"
    in cytosol
~~~~~~~~
```

Figure S1. Bentopy input file structure (*`.bent`*). *Illustrative input file for the JCVI-Syn3A minimal cell model (simplified). The file comprises five sections:* ***General*** *(packing parameters and random seed),* ***Space*** *(simulation box dimensions, voxel resolution),* ***Includes*** *(topology file includes),* ***Compartments*** *(compartment definitions), and* ***Segments*** *(individual molecular species with structure paths, copy numbers / concentrations, compartment assignments, placement rules, and optional tags). Omitted segments are indicated with a wave.*

Packing algorithm

Packing operates through placing molecules sequentially according to a heuristic for their packing difficulty. Before packing begins, all molecules are loaded and ordered according to the heuristic. By default, a ranking based on the geometric moment of inertia ($\mu = \sum_{i \in 0..N} r_i^2$) is used, prioritizing placement of extended molecules that become difficult to pack as spaces fill. Alternative heuristics are also available or the input order can be maintained. Spatial masks defined in the input file are computed from analytical functions or loaded from files, with composite masks generated through boolean operations (union, intersection, negation).

To track available space, the placement procedure uses voxel maps storing the occupancy of the packing volume. A global map accumulates all successfully placed molecules throughout the entire packing procedure and persists across all segments. For each molecule-type, a session map is constructed by overlaying the molecules's assigned compartment mask(s) onto the global, then marking voxels excluded by any placement constraints as inaccessible. This session map defines the valid placement region for the current molecule: voxels must be both within the allowed compartment(s) and unoccupied by previously placed structures. A cache of accessible voxel positions is precomputed from the session background to enable efficient random sampling.

Target copy numbers are determined from specified concentrations (calculated using the accessible volume) or taken directly from the input file. For each placement attempt, the molecule is randomly rotated (respecting any specified constraints) and voxelized. A random position is sampled from the accessible voxel cache, and the voxelized structure is checked against the session background through bitwise comparisons. Any voxel overlap results in rejection. Successful placements update both the global and session backgrounds, and the position and rotation are recorded. If the maximum placement attempts are exhausted, the algorithm proceeds to the next molecule type.

The output file, also referred to as the placement list, stores position and rotation data for all successfully placed molecules in a lightweight, instance-based format. This compact representation serves as input to the subsequent render step, which generates simulation coordinate and topology files.

Solvation algorithm

Solvating large structures with a template-stamping approach brings two main challenges: (1) The number of checks required to determine if a solvent residue may be placed without colliding with the existing structure grows with the number of input structure particles. (2) Storing the accepted solvent placements may require a large amount of system memory.

To address problem (1), we use a spatial partitioning scheme making collision detection tractable for large systems. The coordinates of the input structure are distributed over a spatially indexed array of lists, termed Cookies. Each Cookie contains the structure particles within a

specific spatial region that must be considered when checking for solvent–structure collisions in that region. This spatial lookup table accelerates collision detection by limiting the number of structure particles that must be checked for each solvent placement. Problem (2) is addressed through a bitmap-based representation where each solvent particle is represented as a single bit describing whether it has been accepted for placement. This bitmap, called the PlaceMap, stores placement decisions with minimal memory overhead.

The solvation procedure uses these two data structures together. The algorithm iterates over spatial regions corresponding to each Cookie. For each potential solvent placement, structure particles from the corresponding Cookie are checked for collisions. When a structure and solvent particle are within collision distance, the corresponding bit in the PlaceMap is marked as occupied, rejecting that solvent placement.

Before writing output, the algorithm can optionally substitute some accepted solvent particles with different bead types (e.g., replacing water with ions to achieve target concentrations). The solvent coordinates are first written to disk, followed by any substituted species. A topology file for the solvent structures is generated and can be appended to the input structure topology file.

Showcase models

The construction of all models was performed using a workstation with a 12 thread CPU (Intel® Xeon® W-2135 Processor, 8.25M Cache, 6 cores, 3.70 GHz) and 32 GiB DDR4 memory.

JCVI-Syn3A minimal cell

Model composition

The JCVI-Syn3A minimal cell model integrates multiple cellular components based on experimental data. The complete composition is summarized in Table S1. The cytoplasmic proteins consists of 7,193 molecules representing the 452 protein-coding genes of the JCVI-Syn3A genome, with copy numbers derived from the proteomics dataset of Thornburg et al., 2022. Proteins are categorized into cytosolic (381 molecules) and membrane-peripheral (46 molecules) classes based on localization annotations, enabling compartment-aware and proximity-biased placement during packing. Proteins were placed individually rather than as pre-assembled complexes, meaning that multi-protein assemblies such as ribosomes were not included.

The metabolome comprises 34,693 molecules including all 20 standard amino acids and 9 common cofactors. While this represents a simplified composition restricted to species with available Martini 2 parameterizations, it captures the major metabolite classes essential for cellular function. The metabolite concentrations were taken directly from experimental measurements.

The transcriptome is represented by 496 RNA molecules covering all expressed genes. This includes the four different types of RNA: messenger RNA (mRNA), non-coding RNA (ncRNA), ribosomal RNA (rRNA), and transfer RNA (tRNA). Copy numbers for each RNA species were determined through a constraint-based approach: the total RNA mass matches experimental measurements while relative abundances preserve gene expression ratios from transcriptomics data. The model incorporates pre-built structures, the chromosome and cell envelope, that define the template for our model assembly.

Table S1. JCVI-Syn3A minimal cell model composition

Component	Types	Counts	Sources
**Cytoplasmic proteins**	427	7,193	Thornburg et al., 2022
Cytosolic	381	627	
Membrane-peripheral	46	6,566	
**RNA**	496 (mRNA:458, rRNA:6, tRNA:30, ncRNA:2)	554	Breuer et al., 2019
**Metabolites**	29	34,693	Thornburg et al., 2022
Amino acids	20	23,030	
Cofactors	9	11,663	
**Ions**	2 (Na^+, Cl^-)	2,405,403	
**Cell Envelope**			
Lipids	5 (DOPG:,DPSM, CHOL, CDL2, POPC)	409,089	Justice et al., 2024
Membrane proteins	93	898	Stevens et al., 2023
**Chromosome**	1	1	Gilbert et al., 2023
**Total molecules**	1053	3,388,830	
**Total particles (unsolvated)**	16,215,909		
**Total particles (solvated)**	86,762,148		

Structure preparation

All protein structures were prepared following a standardized protocol for Martini 2. Protein sequences were obtained from the JCVI-Syn3A genome (GenBank: CP016816.2). AlphaFold3 (Abramson et al., 2024) was used to predict structures for all 452 proteins. The atomistic structures were converted to Martini 2 coarse-grained representations using Martinize2 with default parameters and an elastic network (force constant 500 kJ mol^{-1} nm^{-2}, cutoff 0.7 nm) to maintain the secondary structure (Kroon et al., 2024). RNA structures representing the complete JCVI-Syn3A transcriptome were generated using Polyply from sequence information (Grünewald et al., 2022). Polyply generated both Martini 2 parameters and initial coordinates for all RNA molecules. Additionally, an elastic network (force constant 500 kJ mol^{-1} nm^{-2}, cutoff 1.2 nm) was applied to stabilize the RNA structures during simulation. The metabolite models were generated from their itp files using Polyply. Each coarse-grained model underwent 100 ns equilibration in 150 mM NaCl solution to relax the model before packing.

Packing configuration

The packing procedure began by merging the pre-built chromosome and cell envelope structures using *bentopy merge*, producing a combined template structure that defines the geometric constraints for cytosolic placement. *Bentopy* mask analyzed the template structure and detected three compartments with the hierarchy ($0 \supset 1 \supset 2$), labeling them from outermost to innermost as: compartment 0 (extracellular space, 735,000 nm^3), compartment 1 (cell membrane and chromosome, 108,000 nm^3), and compartment 2 (intracellular cytoplasmic volume, 381,000 nm^3). The cell membrane and chromosome were detected as a single compartment, due to close contacts between both structures. Compartment 2, representing the accessible cytoplasmic volume, was established as the packing region. A mask at 0.5 nm resolution defined the accessible cytosolic volume. To implement proximity-based placement rules, a separate voxel mask at 0.5 nm resolution was generated specifically representing the membrane geometry. This membrane mask was derived from the cell envelope structure prior to merging, enabling spatial biasing of molecular placement relative to the membrane independently of the chromosome.

Spatial constraints were implemented through *bentopy*'s rule-based placement system to reflect experimentally-determined localization patterns. Membrane-peripheral proteins were constrained to regions within 15 nm of the envelope using the proximity rule "is_closer_to membrane 15.0", which evaluates distances to the membrane mask. This 15 nm threshold was selected to encompass the largest membrane-peripheral protein in the dataset, ensuring all membrane-associated proteins could be placed within this spatial constraint while maintaining computational efficiency. All other molecules were uniformly distributed throughout the entire cytoplasmic volume without additional spatial restrictions, reflecting their expected distribution throughout the cell.

The packing algorithm (*bentopy pack*) proceeded through molecules in order of decreasing rotational moment of inertia. For each molecule, the algorithm sampled random positions within the permitted mask region and random orientations, accepting placements that exhibited no

voxel overlap with previously placed molecules. If a collision was detected, the algorithm retried the molecule placement up to 50,000 times before moving to the next molecule. The packing procedure completed in 4 minutes, successfully placing 99.7% of requested molecules (41,835 of 41,886). The unpacked fraction consisted exclusively of the largest RNA polymers, reflecting the geometric limitations of rigid-body packing for highly elongated polymers in crowded molecular environments. The challenge of packing long polymers was increased due to the relatively small size of the cell and cytosolic space. The packing output was stored as a placement list, providing a complete specification of the packed system in only 2.3 MB.

Simulation setup

The placement list was converted to coordinate and topology files using *bentopy render*, generating structure files for all placed cytosolic components (106 MB). These were merged with the template structure using *bentopy merge*, producing an unsolvated cell model comprising 16 million coarse-grained beads in a cubic simulation box (214 × 214 × 214 nm^3). The model was solvated using *bentopy solvate*, filling the remaining accessible volume with Martini 2 water beads. Ion placement occurred during solvation, adding sodium and chloride ions to achieve 150 mM NaCl plus additional counterions for charge neutrality. The final solvated model contains 86 million coarse-grained beads (645 MB), representing more than 1 billion atoms at atomistic resolution.

Mitochondrial compartments

Model composition

The mitochondrion model integrates experimentally-derived membrane geometry with compartment-specific solute compositions. The complete composition is summarized in Table S2. The template of the model is a cryo-ET structure of a mouse cerebellum neuron mitochondrion, converted to a Martini 2 lipid bilayer membrane spanning 344 × 796 × 416 nm^3. The soluble protein components span two distinct compartments: the intermembrane space and cristae volume (IMS) and mitochondrial matrix. For each compartment, we identified ten highly abundant soluble proteins based on compartment-specific concentrations from the MitoCoP quantitative mitochondrial proteome dataset (Morgenstern et al., 2021). Within each compartment, protein concentrations were rescaled to reach or exceed estimated protein densities, while maintaining the experimental stoichiometric ratios.

The metabolome in the IMS comprises 828,728 molecules including all 20 standard amino acids and 9 common cofactors. While this represents a simplified composition restricted to species with available Martini 2 parameterizations, it captures the major metabolite classes essential for cellular function. Metabolite concentrations are derived from human mitochondrial metabolomics data. For metabolites with compartment-specific measurements, mitochondrial values were assigned to the matrix and whole-cell values to the IMS. The concentrations of available metabolite species were scaled to match total measured metabolite concentrations while preserving experimental stoichiometries. This yields total metabolite concentrations of 114 mM

in the IMS and 7 mM in the matrix, a 16-fold difference reflecting their distinct metabolic environments.

The RNA component comprises five representative RNA strands at approximate concentrations, restricted to the mitochondrial matrix. While this represents a simplified transcriptome, it captures the presence of RNA molecules within the matrix environment. RNA structures and Martini 2 parameters were generated using Polyply (Grünewald et al., 2022).

Table S2. Mitochondrial compartments model composition

Component	Types	Counts	Sources
**Membrane**			Garcia et al., 2019
Lipids	1	6,702,549	
**IMS**			
Proteins	9	4,694	Morgenstern et al., 2021
Metabolites	28	828,728	Chen et al., 2016
**Matrix**			
Proteins	10	11,922	Morgenstern et al., 2021
Metabolites	17	70,026	Chen et al., 2016
RNA	5	412	
**Ions**	2 (Na^+, Cl^-)	21,914,994	
**Total molecules**	55 (unique sps.)	29,533,325	
**Total particles (unsolvated)**	121,731,991		
**Total particles (solvated)**	985,183,613		

Structure preparation

The mitochondrial membrane structure was derived from cryo-ET of a mouse cerebellum neuron mitochondrion, providing a triangulated surface mesh representing both outer and inner mitochondrial membranes with cristae invaginations. The surface mesh was converted to a Martini 2 lipid bilayer using TS2CG. Four successive pointillism steps were performed to increase the resolution of the mesh. From this geometry, a pure POPC membrane was built with a 2 nm bilayer thickness, 0.64 nm^2 area per lipid, and 0.2 nm bond length. The resulting membranes contain 6.7 million lipids (80 million coarse-grained beads) spanning 344 × 796 × 416 nm^3. The membrane underwent multi-step equilibration. Soft-core energy minimization was performed over 15 steps in double precision with lambda 0.1, applying soft-core potentials for both Van der Waals and Coulomb interactions. This was followed by conventional energy minimization for 1000 steps in double precision, then final minimization in single precision, ensuring the membrane structure was relaxed before cytosolic packing.

Protein structures for IMS and matrix compartments were obtained from the Protein Data Bank where available or predicted using AlphaFold2. All structures were prepared by removing non-protein components and adjusting titration states to pH 7.0 using pdb2pqr with PROPKA (Jurrus et al., 2018). The atomistic structures were converted to Martini 2 representations using Martinize2 with an elastic network (force constant 500 kJ mol^{-1} nm^{-2}, lower and upper bounds 0.5 and 1.0 nm) (Kroon et al., 2024). Each coarse-grained protein was equilibrated individually before packing. The protocol consisted of energy minimization under vacuum followed by a solvation with Martini water and 10% antifreeze water. Additionally, during the solvation step 150 mM NaCl was added plus additional counterions for charge neutrality, followed by another energy minimization. A 1 ns equilibration at a 10 fs timestep, and 50 ns production at a 20 fs timestep were performed for each structure. Metabolite models were generated from their parameter files using Polyply.

Packing configuration

Automated compartment detection (*bentopy mask*) analyzed the equilibrated membrane structure to identify distinct cytosolic volumes. The algorithm processed the lipid bilayer connectivity to delineate two compartments based on membrane topology: the IMS (volume between outer and inner membranes) and matrix (volume enclosed by inner membrane). Voxel masks at 0.5 nm resolution were generated for each compartment, representing 12 × 10^6 nm^3 (IMS) and 16 × 10^6 nm^3 (matrix).

The packing input files specified the appropriate compartment mask, protein structures with target copy numbers, and metabolite concentrations for each region. All molecules were assigned to their full compartment volumes without additional spatial restrictions, reflecting expected uniform distribution within each compartment. The packing algorithm (*bentopy pack*) processed molecules in order of decreasing rotational moment of inertia for each compartment independently. For each molecule, the algorithm sampled random positions within the assigned compartment mask and random orientations, accepting placements without voxel overlap with previously placed molecules. If a collision was detected, the algorithm retried up to 10,000 times

before moving to the next molecule. The packing procedure completed in 4 minutes, successfully placing 99.9% of requested molecules (915,782 of 916,812). The compartment-specific approach ensures molecules are placed only in designated volumes, automatically respecting membrane boundaries without explicit collision checking against the membrane structure. The packing outputs were stored as a placement list (142 MB).

Simulation setup

The placement list was converted to coordinate and topology files using *bentopy render*. These were then merged with the equilibrated membrane structure using *bentopy merge*, producing a combined unsolvated mitochondrion model. The unsolvated model comprises 122 million coarse-grained beads: 80 million from the POPC lipids in the membrane, and 42 million from packed cytosolic components. The model was solvated using *bentopy solvate*, filling the remaining accessible volume in both compartments with Martini 2 water containing 10% antifreeze beads. Ion placement occurred during solvation, adding sodium and chloride ions to achieve 150 mM NaCl plus additional counterions for charge neutrality (a -1351044 charge was neutralized by 1351044 sodium ions). The final solvated model contains 958 million coarse-grained beads (44 GB), representing more than 10 billion atoms at atomistic resolution.

SARS-CoV-2 aerosol

Model composition

The SARS-CoV-2 aerosol model reproduces the system described by Dommer et al.(Dommer et al., 2023), representing a complete virion embedded in deep lung fluid within a respiratory aerosol droplet. The complete composition is summarized in Table S3. The virion structure is obtained from previous work and includes all structural proteins (29 spike proteins, 360 M-protein dimers, and 4 E-protein pentamers) and the viral envelope (POPC, POPE, POPI, POPS, CHOL). This pre-built structure serves as the template for subsequent packing. The lung fluid composition is based on the composition of artificial saliva and surrogate deep lung fluid. The lipid components are DPPC (6.5 mM), DPPG (0.69 mM), and CHOL (0.26 mM). Protein components comprise 199 copies of human serum albumin and 480 copies of mucin peptides across three different variants. Physiological ion concentrations include Ca^{2+} (1.4 mM), Mg^{2+} (0.8 mM), and Na^{+} (142 mM). The three mucin glycoproteins included have full O-glycosylation patterns based on experimentally-determined protein sequences and glycosylation sites (Kearns et al., 2024).

Table S3. ***SARS-CoV-2 aerosol*** **model composition**

Component	Types	Counts	Sources
**Virion**			Dommer et al., 2023
Proteins	3	393	
Lipids	5<br>(POPC / POPE / POPI / POPS)	60,046	
**Aerosol**			Dommer et al., 2023
Lipids	3<br>(DPPC, DPPG, CHOL)	12,326	
Proteins	2	679	
**Ions**	5	1,777,273	
**Total molecules**	16	23,566	
**Total atoms (unsolvated)**	67,186,844		
**Total atoms (solvated)**	1,018,236,381		

Structure preparation

The virion structure and all lung fluid components were obtained from the Dommer et al. model. Topology files were converted from NAMD to GROMACS format using CHARMM-GUI and the CHARMM36m force field (Huang et al., 2017; Jo et al., 2008).

Packing configuration

The packing procedure required defining spatial constraints representing both the virion exclusion and the aerosol droplet boundary. A voxel mask at 0.5 nm resolution was generated from the complete virion structure, identifying all voxels occupied by the virion to create an exclusion region. To define the aerosol boundary, a second mask representing a 270 nm diameter sphere centered on the virion was created. These masks were combined with a boolean AND operation to define the final packing region: space inside the spherical boundary but outside the virion structure.

Spatial placement rules were implemented for distinct molecule types. Lung fluid components (lipids, proteins, standard ions) were assigned to the combined mask representing the accessible aerosol volume. We additionally implemented targeted ion placement using the proximity-based placement rules. An additional mask was generated representing only the M-protein dimers from the virion structure. The 360 M-protein dimers carry a total intravirion charge of -5,696, requiring neutralization. Rather than distributing Na^+ counter-ions throughout the system, a proximity-based packing rule was used to place the counter ions within 5 nm of the M-proteins. This mask guided placement of counter-ions near their target proteins, dramatically improving the initial electrostatic environment compared to random ion placement.

The packing algorithm (*bentopy pack*) processed molecules in order of decreasing rotational moment of inertia for each compartment independently. For each molecule, the algorithm sampled random positions within the assigned compartment mask and random orientations, accepting placements without voxel overlap with previously placed molecules. If a collision was detected, the algorithm retried up to 5,000 times before moving to the next molecule. The packing procedure completed in 9 seconds, successfully placing 100% of the 23,837 requested molecules. The packing output was stored as a placement list, providing a complete specification of the packed system in only 99 MB.

Simulation setup

The placement list was converted to coordinate and topology files using *bentopy render*. These were then merged with the virion structure using *bentopy merge*, producing a combined unsolvated SARS-CoV-2 aerosol model. The unsolvated model comprises 67 million atoms: 14 million from the virion model, and 53 million from packed lung fluid components. The model was solvated using *bentopy solvate*, filling the remaining accessible volume with TIP3P water molecules, ignoring collisions with ions placed by *bentopy pack*. Water residues outside the aerosol sphere were cut. The final solvated model contains more than 1 billion atoms and results in a 46 GB structure (.gro) file.

Citations

Abraham, M. J., Murtola, T., Schulz, R., et al. (2015). GROMACS: High performance molecular simulations through multi-level parallelism from laptops to supercomputers. *SoftwareX*, *1–2*, 19–25. https://doi.org/10.1016/j.softx.2015.06.001

Abramson, J., Adler, J., Dunger, J., et al. (2024). Accurate structure prediction of biomolecular interactions with AlphaFold 3. *Nature*, *630*(8016), 493–500. https://doi.org/10.1038/s41586-024-07487-w

Bernetti, M., & Bussi, G. (2020). Pressure control using stochastic cell rescaling. *The Journal of Chemical Physics*, *153*(11), 114107. https://doi.org/10.1063/5.0020514

Breuer, M., Earnest, T. M., Merryman, C., et al. (2019). Essential metabolism for a minimal cell. *eLife*, *8*, e36842. https://doi.org/10.7554/eLife.36842

Bussi, G., Donadio, D., & Parrinello, M. (2007). Canonical sampling through velocity rescaling. *The Journal of Chemical Physics*, *126*(1), 014101. https://doi.org/10.1063/1.2408420

Chen, W. W., Freinkman, E., Wang, T., Birsoy, K., & Sabatini, D. M. (2016). Absolute Quantification of Matrix Metabolites Reveals the Dynamics of Mitochondrial Metabolism. *Cell*, *166*(5), 1324-1337.e11. https://doi.org/10.1016/j.cell.2016.07.040

De Jong, D. H., Baoukina, S., Ingólfsson, H. I., & Marrink, S. J. (2016). Martini straight: Boosting performance using a shorter cutoff and GPUs. *Computer Physics Communications*, *199*, 1–7. https://doi.org/10.1016/j.cpc.2015.09.014

Dommer, A., Casalino, L., Kearns, F., et al. (2023). #COVIDisAirborne: AI-enabled multiscale computational microscopy of delta SARS-CoV-2 in a respiratory aerosol. *The International Journal of High Performance Computing Applications*, *37*(1), 28–44. https://doi.org/10.1177/10943420221128233

Essmann, U., Perera, L., Berkowitz, M. L., Darden, T., Lee, H., & Pedersen, L. G. (1995). A smooth particle mesh Ewald method. *The Journal of Chemical Physics*, *103*(19),

8577–8593. https://doi.org/10.1063/1.470117

Garcia, G. C., Bartol, T. M., Phan, S., et al. (2019). Mitochondrial morphology provides a mechanism for energy buffering at synapses. *Scientific Reports*, *9*(1), 18306. https://doi.org/10.1038/s41598-019-54159-1

Gilbert, B. R., Thornburg, Z. R., Brier, T. A., et al. (2023). Dynamics of chromosome organization in a minimal bacterial cell. *Frontiers in Cell and Developmental Biology*, *11*. https://doi.org/10.3389/fcell.2023.1214962

Grünewald, F., Alessandri, R., Kroon, P. C., Monticelli, L., Souza, P. C. T., & Marrink, S. J. (2022). Polyply; a python suite for facilitating simulations of macromolecules and nanomaterials. *Nature Communications*, *13*(1), Article 1. https://doi.org/10.1038/s41467-021-27627-4

Huang, J., Rauscher, S., Nawrocki, G., et al. (2017). CHARMM36m: An improved force field for folded and intrinsically disordered proteins. *Nature Methods*, *14*(1), 71–73. https://doi.org/10.1038/nmeth.4067

Jo, S., Kim, T., Iyer, V. G., & Im, W. (2008). CHARMM-GUI: A web-based graphical user interface for CHARMM. *Journal of Computational Chemistry*, *29*(11), 1859–1865. https://doi.org/10.1002/jcc.20945

Jurrus, E., Engel, D., Star, K., et al. (2018). Improvements to the APBS biomolecular solvation software suite. *Protein Science*, *27*(1), 112–128. https://doi.org/10.1002/pro.3280

Justice, I., Kiesel, P., Safronova, N., von Appen, A., & Saenz, J. P. (2024). A tuneable minimal cell membrane reveals that two lipid species suffice for life. *Nature Communications*, *15*(1), 9679. https://doi.org/10.1038/s41467-024-53975-y

Kearns, F. L., Rosenfeld, M. A., & Amaro, R. E. (2024). Breaking Down the Bottlebrush: Atomically Detailed Structural Dynamics of Mucins. *Journal of Chemical Information and Modeling*, *64*(20), 7949–7965. https://doi.org/10.1021/acs.jcim.4c00613

Kim, H., Fábián, B., & Hummer, G. (2023). Neighbor List Artifacts in Molecular Dynamics

Simulations. *Journal of Chemical Theory and Computation*, *19*(23), 8919–8929. https://doi.org/10.1021/acs.jctc.3c00777

Kroon, P. C., Grunewald, F., Barnoud, J., et al. (2024). Martinize2 and Vermouth: Unified Framework for Topology Generation. *eLife*, *12*. https://doi.org/10.7554/eLife.90627.2

Marrink-lab/bentopy. (2026). Reference for bent. Github. Retrieved January 22, 2026, from https://github.com/marrink-lab/bentopy/wiki/Reference-for-bent

Morgenstern, M., Peikert, C. D., Lübbert, P., et al. (2021). Quantitative high-confidence human mitochondrial proteome and its dynamics in cellular context. *Cell Metabolism*, *33*(12), 2464-2483.e18. https://doi.org/10.1016/j.cmet.2021.11.001

Stevens, J. A., Grünewald, F., van Tilburg, P. A. M., et al. (2023). Molecular dynamics simulation of an entire cell. *Frontiers in Chemistry*, *11*. https://doi.org/10.3389/fchem.2023.1106495

Thornburg, Z. R., Bianchi, D. M., Brier, T. A., et al. (2022). Fundamental behaviors emerge from simulations of a living minimal cell. *Cell*, *185*(2), 345-360.e28. https://doi.org/10.1016/j.cell.2021.12.025
